# Relationship between chorioamnionitis or funisitis and lung injury among preterm infants: meta-analysis involved 16 observational studies with 68,397 participants

**DOI:** 10.1186/s12887-024-04626-0

**Published:** 2024-03-05

**Authors:** Wen-li Liu, Yao Zhou, Chao Zhang, Jun Chen, Xu-feng Yin, Feng-xia Zhou, Shao-jun Chen

**Affiliations:** 1 Department of Neonatology, Sinopharm Dongfeng General Hospital, Hubei University of Medicine, Shiyan, Hubei China; 2 Department of Surgery, School of Medicine, Jianghan University, Wuhan, Hubei China; 3 Center for Evidence-Based Medicine and Clinical Research, Taihe Hospital, Hubei University of Medicine, Shiyan, Hubei China; 4 Sinopharm Dongfeng General Hospital, Hubei University of Medicine, Shiyan, 442008 Hubei China

**Keywords:** Chorioamnionitis, Funisitis, Preterm infants, Lung injury, Neonatal respiratory distress syndrome, Bronchopulmonary dysplasia, Systematic review, Meta-analysis

## Abstract

**Background:**

Chorioamnionitis (CA) can cause multiple organ injuries in premature neonates, particularly to the lungs. Different opinions exist regarding the impact of intrauterine inflammation on neonatal respiratory distress syndrome (NRDS) and bronchopulmonary dysplasia (BPD). We aim to systematically review the relationship between CA or Funisitis (FV) and lung injury among preterm infants.

**Methods:**

We electronically searched PubMed, EMbase, the Cochrane library, CNKI, and CMB for cohort studies from their inception to March 15, 2023. Two reviewers independently screened literature, gathered data, and did NOS scale of included studies. The meta-analysis was performed using RevMan 5.3.

**Results:**

Sixteen observational studies including 68,397 patients were collected. Meta-analysis showed CA or FV increased the lung injury risk (OR = 1.43, 95%CI: 1.06–1.92). Except for histological chorioamnionitis (HCA) (OR = 0.72, 95%CI: 0.57–0.90), neither clinical chorioamnionitis (CCA) (OR = 1.86, 95%CI: 0.93–3.72) nor FV (OR = 1.23, 95%CI: 0.48–3.15) nor HCA with FV (OR = 1.85, 95%CI: 0.15–22.63) had statistical significance in NRDS incidence. As a result of stratification by grade of HCA, HCA (II) has a significant association with decreased incidence of NRDS (OR = 0.48, 95%CI: 0.35–0.65). In terms of BPD, there is a positive correlation between BPD and CA/FV (CA: OR = 3.18, 95%CI: 1.68–6.03; FV: OR = 6.36, 95%CI: 2.45–16.52). Among CA, HCA was positively associated with BPD (OR = 2.70, 95%CI: 2.38–3.07), whereas CCA was not associated with BPD (OR = 2.77, 95%CI: 0.68–11.21). HCA and moderate to severe BPD (OR = 25.38, 95%CI: 7.13–90.32) showed a positive correlation, while mild BPD (OR = 2.29, 95%CI: 0.99–5.31) did not.

**Conclusion:**

Currently, evidence suggests that CA or FV increases the lung injury incidence in premature infants. For different types of CA and FV, HCA can increase the incidence of BPD while decreasing the incidence of NRDS. And this “protective effect” only applies to infants under 32 weeks of age. Regarding lung injury severity, only moderate to severe cases of BPD were positively correlated with CA.

**Supplementary Information:**

The online version contains supplementary material available at 10.1186/s12887-024-04626-0.

## Background

Premature infants can be exposed to intrauterine infections up to 40%-70% [1, 2], of which CA is the leading cause of spontaneous preterm birth [3, 4]. An estimated 2% of pregnant women around the world are affected by CA, which refers to an infection of the amniotic fluid, placenta, or fetus caused by pathogenic bacteria [5, 6]. It is defined as the infiltration of multinucleate cells into the amniotic membrane and the chorionic membrane, and if the infection or inflammation processes involving the umbilical cord (umbilical vein, umbilical artery, and the Wharton’s jelly) are referred to as acute funisitis (FV) [7, 8]. After birth of premature infants, CA can cause multiple organ injuries, including lung, brain, gastrointestinal injuries, and the outcome is worse when combined with FV, which constitutes fetal inflammation or fetal inflammatory response syndrome [5, 9, 10]. CA has been found to be associated with a wide range of common postnatal diseases in premature infants, including respiratory distress syndrome (RDS), bronchiolar pulmonary dysplasia (BPD), intraventral hemorrhage and necrotizing enterocolitis (NEC), with the lung being the most easily affected part [11].

In premature infants, neonatal respiratory distress syndrome (NRDS) is a common respiratory condition caused by the decreased production or increased destruction of pulmonary surfactant (PS) secreted by alveolar type II epithelial cells. This condition is characterized by progressive and aggravated respiratory distress [12, 13] and is frequently associated with respiratory disease and death in newborns [14]. According to statistics, the overall prevalence rate of neonatal ARDS is 1.5%, and the overall mortality rate is as high as 17% ~ 24% [15]. Infection (both internal and external to the lung), acidosis, asphyxia [16] preterm birth, diabetic mothers, and insufficient secretion of primary PS are some of the causes of NRDS [17]. However, some studies have discovered fetal lung development can be aided by intrauterine inflammation [18–20].

At present, there are only three pertinent systematic reviews and meta-analyses [21–23] on the impact of CA and FV on lung injuries in premature infants. Some similar subjects have been conducting in China, while there are no relevant reviews and meta-analyses. Glucocorticoids possess strong anti-inflammatory properties. Early intravenous administration of glucocorticoids inhibits the inflammatory response in the lungs of extremely premature infants [24]. As a result, to improve clinical workers' attention to such premature infants and invite neonatal intensive care unit (NICU) consultation as soon as possible after birth, we still require a lot of clinical research data to further explore the relationship between CA or FV and lung injury in premature infants, thereby allowing for early intervention. As a result, it can lower disease incidence, reduce disease severity, improve the prognosis for children, and serve as a theoretical basis for reducing the burden on families and conserving social resources.

## Methods

A systematic review of cohort study was conducted according to the Cochrane Handbook for Systematic Reviews of Interventions [25]. The protocol was registered on PROSPERO, the international database of prospectively registered systematic reviews. The systematic review was reported using the Preferred Reporting Items for Systematic Reviews and Meta-Analyses statement [26].

### Search strategy

Original research on the association between chorioamnionitis or funisitis and Lung Injury among Preterm Infants was retrieved from PubMed, EMbase, the Cochrane library, CNKI and CMB, from inception to March 15, 2023. The retrieval formula for this study was as follows: ((Chorioamnionitis) OR (Chorioamnionitides) OR (Amnionitis) OR (Amnionitides) OR (Funisitis) OR (Funisitides)) AND ((Neonatal respiratory distress syndrome) OR (Infantile Respiratory Distress Syndrome) OR (Neonatal Respiratory Distress Syndrome) OR (Respiratory Distress Syndrome, Infant) OR (bronchopulmonary dysplasia) OR (Dysplasia, Bronchopulmonary) OR (Lung Injury) OR (injuries lung) OR (injury lung) OR (pulmonary injury) OR (injuries pulmonary) OR (injury pulmonary) OR (pulmonary injuries) OR (lung injuries) OR (chronic lung injury) OR (chronic lung injuries) OR (injuries chronic) OR (lung injury chronic)). Additional strategies for identifying studies included a manual review of reference lists from key articles that fulfilled our eligibility criteria as well as other systematic reviews on CA, use of the “related articles” feature in PubMed, and use of the “cited by” tool in Web of Science. No language limit was applied. Narrative reviews, systematic reviews, letters, editorials, case–control studies and commentaries were excluded but were read to identify potential additional studies.

### Eligibility and excluded criteria

The following criteria were used to select studies for inclusion in the meta-analysis: (1) a population under 37 weeks of age; (2) single pregnancy; (3) exposure to CA/FV; (4) the non-exposed group is non-CA/FV (N-CCA/N-HCA/N-FV); (5) the outcome was lung injury, including RDS and BPD; (6) provide definitions of outcomes (RDS/BPD); (7) provide primary data to assess the relationship between CA/FV exposure and lung injury (RDS/BPD); (8) cohort study.

Excluded criteria: (1) the included population was not preterm infants; (2) twins or multiple pregnancies; (3) pregnancy status was not specified; (4) infants with extremely low birth weight without certainty of preterm birth; (5) the definition of the outcome indicator is not provided; (6) data missing; (7) other researches’ design such as case–control.

### Data extraction

Two of us (LWL, ZY) independently screened the search results and applied inclusion criteria using a structured form to identify relevant studies. In several rounds: by the title alone in the first round, the title and abstract in the second and the full text in the third. Discrepancies were settled through discussion or consultation with a third reviewer (CSJ). Data extraction and quality assessment were carried out by two authors (LWL and ZY) and verified the accuracy and completeness of the data extraction by a second reviewer (ZC). Using a predetermined data extraction form, the following details were extracted from pertinent studies: the citation information, the country of the participants, the average gestational age and weight of each study, the study design, BPD definitions, the number of patients in each exposure group and control group, and other directly extractable data were first extracted for each study. Incidentally, classification and integration were estimated for data not reported directly.

In regard to lung injury, we research two types of diseases: NRDS and BPD. NRDS was diagnosed and graded in accordance with the Paediatric Acute Lung Injury Consensus Conference (PALICC) [27]. And BPD was diagnosed and graded according to the National Institute of Child Health and Human Development (NICHD) [28]. In addition, there are two definitions of BPD, one of which is defined as oxygen dependency at 36 weeks after menstruation (BPD36) and the other as dependency on oxygen supplementation at a corrected age of 28 days (BPD28) [28]. The diagnosis and grading criteria for CA (HCA, CCA) and FV refer to pathological diagnoses [7, 29]. All studies with the same definition were included. Studies that did not explicitly state a definition of CA (HCA/CCA) were excluded from subgroup classification.

### Validity assessment

We independently read and evaluated the quality of the literature included with the Newcastle–Ottawa Scale (NOS) [30]. Each asterisk represents a point, and the total score is the sum of all the points. A total score of 7 or higher was considered high quality research. Studies were excluded if data could not be extracted or obtained by contacting the authors. Duplicate studies were also ignored.

### Statistical analysis

The effect of CA or FV on lung injury was analyzed using the odds ratio (OR) and 95% confidence interval (CI) as an effect measure. Between-study heterogeneity was assessed using Cochran’s Q and I^2^ statistics. Initial analyses with I^2^ ≤ 40% was performed with a fixed-effects model, otherwise, the random effects model is adopted. The potential confounding variables included gestational age, birth weight, the severity of lung injury (BPD (mild, moderate, severe, and moderate or severe) and NRDS), maternal age, antenatal corticosteroids, and the different classification and staging of CA (HCA (I, II, and III) and CCA) were considered the primary sources of heterogeneity, and subgroup analyses were carried out. All statistical analyses were performed with RevMan 5.3. The exposure group was divided into five distinct categories: CA, HCA, CCA, FV, HCA, and FV. The funnel plot was used to evaluate bias, and a portion of the bias was determined by observing the funnel plot’s symmetry.

## Results

### All reference studies

**Fig. 1:**
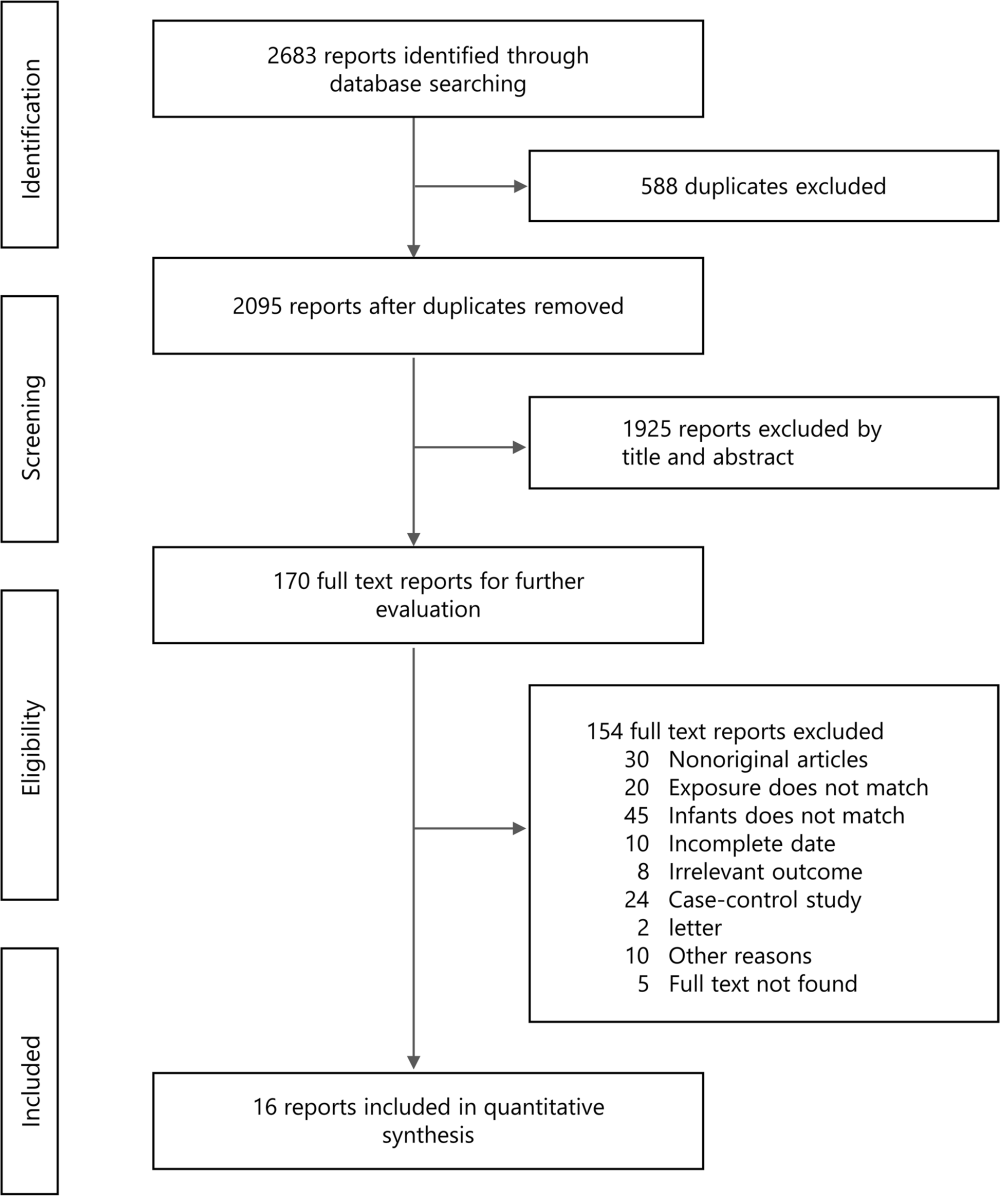
Flow diagram of study selection

We searched a total of 2,683 studies. After initial evaluation, 588 studies were removed for being duplicates, 1,925 for being irrelevant (as determined by reading the title and abstracts), and 154 studies for reasons determined by reading the full text. Ultimately, 16 studies [19, 31–45] (shown in Additional file 1) were identified, involving 68,397 participants. Figure 1 shows a flow diagram of the process.

Among these 16 articles, for RDS outcome in preterm infants, 11 studies [19, 31, 34–38, 40, 42, 43, 45] discuss HCA and its relationship, 2 [32, 39] discuss CCA and its relationship, 4 [19, 33–35] discuss FV and its relationship and 2 [19, 40] discuss HCA with FV and its relationship; for BPD outcome in preterm infants, 7 articles [34, 37, 38, 40, 41, 43, 44] research correlation between BPD and HCA, 2 [32, 39] discuss BPD and CCA; 6 studies [34, 38–41, 44] provided data on BPD28 and 3 studies [32, 37, 43] provided data on BPD36; 1study [44] provided data on mild and moderate to severe BPD. The number of cases in 5 studies [32, 36, 38, 39, 43] exceeds 500; for CA, we quest the influence of the classification and the stage on BPD and RDS in preterm infants. The primary characteristics of the research cited in this document are presented in Additional file 1. 154 studies were excluded from the analysis, as detailed in Additional file 2. The evaluation details and results are presented in Additional file 3. We identify 14 high-quality studies [19, 31–37, 39–42, 44] with a score of 7 or higher from the NOS scale. Moreover, all funnel plots are symmetrical and unbiased.

### Whether CA or FV impact on lung injury in preterm infants

**Fig. 2:**
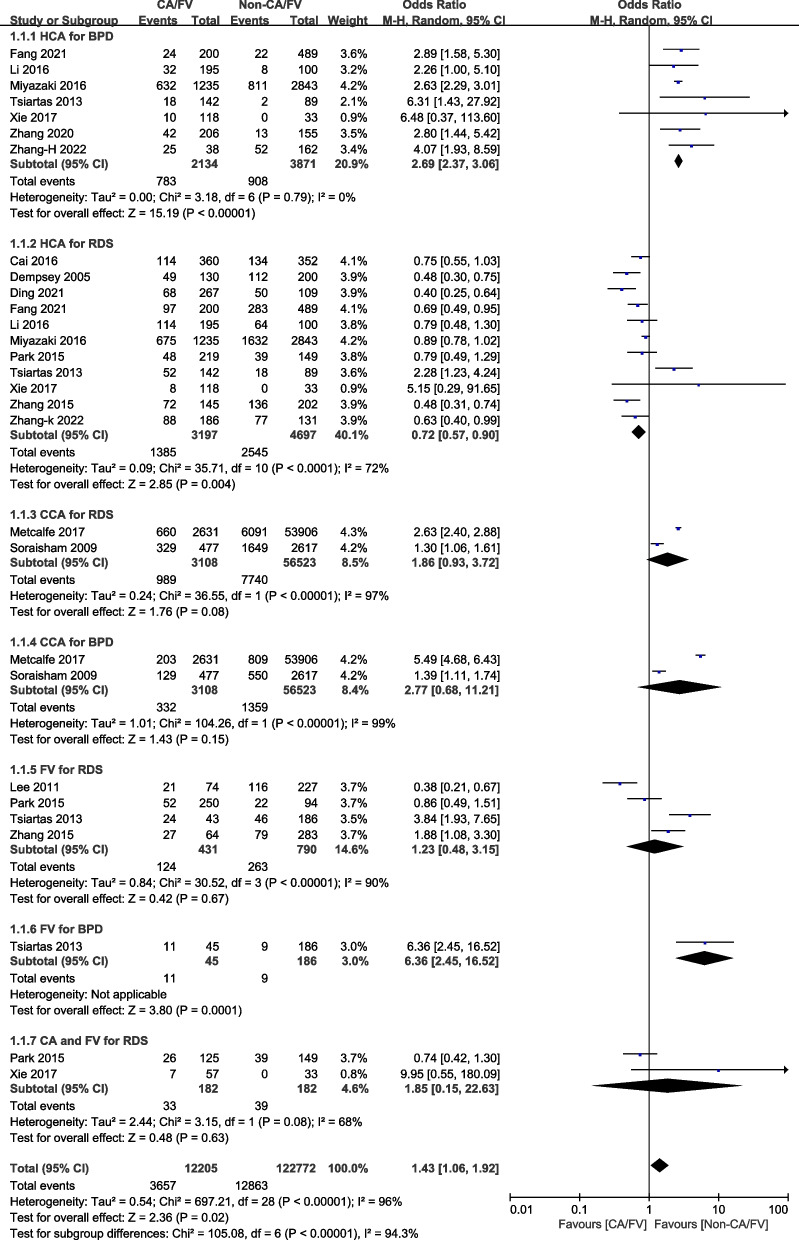
Meta-analysis of the association between Chorioamnionitis or Funisitis and lung injury

A total of 16 studies were enrolled on the relationship between CA or FV and lung injury, with CA (HCA, CCA), FV, HCA and FV as exposure groups, and no CA or FV as non-exposure groups. Comparing to the non-CA/FV, calculations (OR = 1.43, 95%CI: 1.06–1.92, I^2^ = 96%) in Table 1 and Fig. 2 demonstrated that CA or FV were associated with an increased incidence of lung injury in premature infants, particularly in developed countries (OR = 1.56, 95%CI: 1.08–2.27, I^2^ = 96%), and were not statistically significant in developing countries (OR = 1.24, 95%CI: 0.82–1.86, I^2^ = 85%). Two types of cohort studies were studied in this study. Prospective research included 4 studies [19, 34, 40, 45] that showed statistical significance (OR = 1.82, 95%CI: 1.04–13.17, I^2^ = 80%), while retrospective research included 12 studies [31–33, 35–39, 41–44] that showed no statistical significance (OR = 1.30, 95%CI: 0.91–1.85, I^2^ = 97%).

**Table 1:** Subgroup analyses of different outcomes

Outcome indicators	Number of studies	Sample size	OR, 95%CI	P for OR	I^2^	I^2^ for P
Lung Injure(Exposure vs. Non-Exposure)	16	68397	1.43 (1.06,1.92)	0.02	96%	< 0.00001
Retrospective	12	67320	1.30 (0.91,1.85)	0.15	97%	< 0.00001
Prospective	4	1077	1.82 (1.04,3.17)	0.04	80%	< 0.00001
Country developed	7	64949	1.56 (1.08,2.27)	0.02	97%	< 0.00001
Country developing	9	3448	1.24 (0.82,1.86)	0.31	85%	< 0.00001
High quality studies	14	67391	1.49 (1.09,2.03)	0.01	96%	< 0.00001

### NRDS

#### Different types of CA or FV on NRDS

Sixty-seven thousand eight hundred thirty-six participants were included in 14 studies; [19, 31–40, 42, 43, 45] the results (OR = 0.97, 95%CI: 0.67–1.40, I^2^ = 96%) indicated no statistical significance between infants exposed to CA or FV and the incidence of NRDS (Table 2). Separate meta-analyses were also conducted for CA (HCA and CCA), FV and HCA with FV. The results indicated that neither CA (OR = 0.87, 95%CI: 0.57–1.33, I^2^ = 97%) nor FV (OR = 1.23, 95%CI: 0.48–3.15, I^2^ = 90%), nor HCA with FV (OR = 1.85, 95%CI: 0.15–22.63, I^2^ = 68%) were statistically significant. Furthermore, no statistically significant difference was observed between those exposed to CCA and those who were not (OR = 1.86, 95%CI: 0.93–3.72, I^2^ = 97%). Based on the data (OR = 0.72, 95%CI: 0.57–0.90, I^2^ = 72%), exposure to HCA was associated with a reduction in NRDS incidence among preterm infants, with 7 studies [35–37, 40, 42, 43, 45] from developing countries showing statistically significant results (OR = 0.64, 95%CI: 0.55–0.75, I^2^ = 36%), while 4 studies [19, 31, 34, 38] from developed countries were not (OR = 0.9, 95%CI: 0.57–1.42, I^2^ = 82%).

**Table 2:** Subgroup analyses of^a^ NRDS

Outcome indicators	Number of studies	Sample size	OR, 95%CI	P for OR	I^2^	I^2^ for P
^b^CA/^c^FV for NRDS	14	67836	0.97 (0.67,1.40)	0.88	96%	< 0.00001
< 32 weeks	10	10061	0.74 (0.58,0.95)	0.02	79%	< 0.00001
≥ 32 weeks	4	57775	1.65 (0.82,3.32)	0.16	95%	< 0.00001
CA for NRDS	13	67535	0.87 (0.57,1.33)	0.53	97%	< 0.00001
^d^HCA	11	7894	0.72 (0.57,0.90)	0.004	72%	< 0.0001
< 32 weeks	8	6666	0.63 (0.48,0.82)	0.0005	71%	0.001
≥ 32 weeks	3	1238	1.05 (0.57,1.94)	0.87	81%	0.006
HCA(I)	3	978	0.58 (0.31,1.11)	0.1	78%	0.01
HCA(II)	3	955	0.48 (0.35,0.65)	< 0.00001	0%	0.65
HCA(III)	3	743	1.27 (0.64,2.50)	0.5	64%	0.06
Country developed	4	5007	0.9 (0.57,1.42)	0.65	82%	0.0009
Country developing	7	2887	0.64 (0.55,0.75)	< 0.00001	36%	0.15
^e^CCA	2	59631	1.86 (0.93,3.72)	0.08	97%	< 0.00001
FV for NRDS	4	1221	1.23 (0.48,3.15)	0.67	90%	< 0.00001
HCA and FV for NRDS	2	364	1.85 (0.15,22.63)	0.63	68%	0.08

^a^*NRDS* Neonatal respiratory distress syndrome

^b^*CA* Chorioamnionitis

^c^*FV* Funisitis

^d^*HCA* Histological chorioamnionitis

^e^*CCA* Clinical chorioamnionitis

#### Different stages of HCA on NRDS

The viewpoints of HCA for NRDS that early protection, late damage were showed in some studies. We further assembled the data on different stages of HCA for lung injury [19, 36, 42]. When further stratified by grade of HCA, HCA (II) is significantly related to decrease incidence of NRDS (HCA (II): OR = 0.48, 95%CI: 0.35–0.65, I^2^ = 0%), as well as HCA (I and II) (OR = 0.54, 95%CI: 0.39–0.76, I^2^ = 57%). However, the results (HCA (I): OR = 0.58, 95%CI: 0.31–1.11, I^2^ = 78%; HCA (III): OR = 1.27, 95%CI: 0.64–2.50, I^2^ = 64%) showed that HCA (I) and HCA (III) has no obvious protective against NRDS.

#### Gestational age

**Fig. 3:**
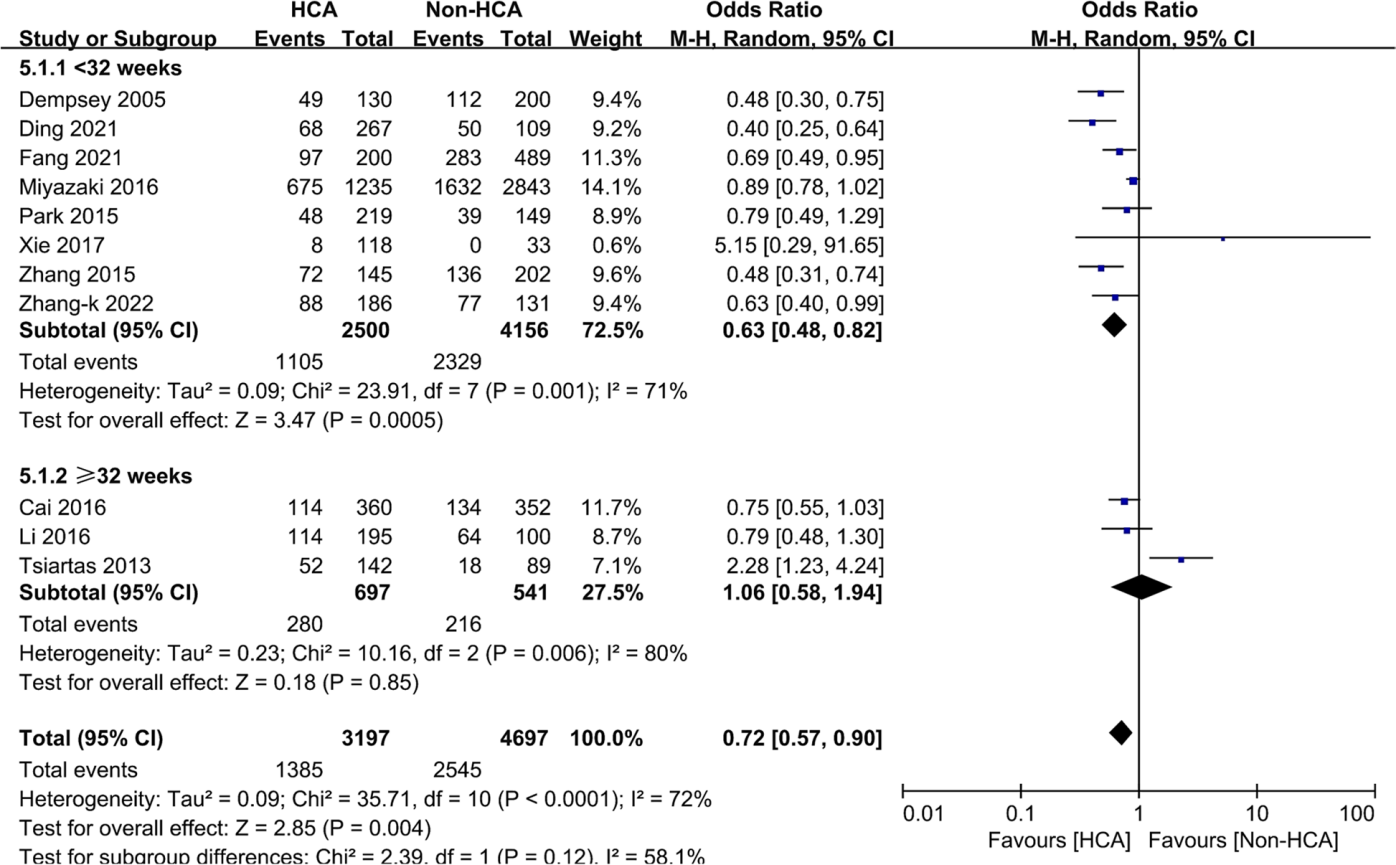
Meta-analysis of the association between HCA and NRDS in premature infants of different gestational ages

As preterm infants < 32 weeks of gestation are in the late tubule or cystic stage, we compared the relationship between HCA and NRDS at < 32 weeks and ≥ 32 weeks (Fig. 3). The results (OR = 0.63, 95%CI: 0.48–0.82, I^2^ = 71%) suggested that exposure to HCA for infants under 32 weeks [19, 31, 35, 40, 42, 43, 45] significantly decreased the incidence of NRDS, but there was no significant difference between infants ≥ 32 weeks [34, 36, 37] (OR = 1.05, 95%CI: 0.57–1.94, I^2^ = 81%).

### BPD

#### Different diagnose of BPD

The definition of BPD has also evolved as research on BPD has progressed. One was diagnosed with oxygen dependency at 36-wk postmenstrual age, and another was diagnosed with dependency on oxygen supplementation at a corrected age of 28 d. We examined whether preterm infants exposed to CA or FV would be at a higher risk of developing BPD in light of the two different definitions of BPD. From the data: A positive association was be found between exposure to CA or FV and BPD28 (OR = 3.83, 95%CI: 2.33–6.3, I^2^ = 90%), and the association remained significant for CA or FV and BPD36 (OR = 1.94, 95%CI: 1.16–3.25, I^2^ = 66%).

#### Different types of CA or FV on BPD

Based on the results shown in Table 3, there is a positive relationship between CA or FV and BPD (OR = 3.21, 95%CI: 2.1–4.93, I^2^ = 92%) regardless of whether infants are exposed to CA (OR = 3.18, 95%CI: 1.68–6.03, I^2^ = 93%) or FV (OR = 6.36, 95%CI: 2.45–16.52) and whether they are born in developed countries (OR = 3.39, 95%CI: 1.83–6.27, I^2^ = 96%) or in developing countries (OR = 2.99, 95%CI: 2.11–4.23, I^2^ = 0%). The prevalence of BPD is significantly associated with infants exposed to HCA (OR = 2.70, 95%CI: 2.38–3.07, I^2^ = 0%), but not with infants exposed to CCA (OR = 2.77, 95%CI: 0.68–11.21, I^2^ = 99%).

**Table 3:** Subgroup analyses of ^a^BPD

Outcome indicators	Number of studies	Sample size	OR, 95%CI	P for OR	I^2^	I^2^ for P
^b^CA/^c^FV for BPD	9	65636	3.21 (2.1,4.93)	< 0.00001	92%	< 0.00001
Country developed	4	63940	3.39 (1.83,6.27)	< 0.0001	96%	< 0.00001
Country developing	5	1696	2.99 (2.11,4.23)	< 0.00001	0%	0.84
< 32 weeks	6	8573	2.47 (1.68,3.65)	< 0.00001	81%	< 0.0001
≥ 32 weeks	3	57063	5.19 (4.41,6.11)	< 0.00001	36%	0.19
BPD28	6	61558	3.83 (2.33,6.3)	< 0.00001	90%	< 0.00001
BPD36	3	4078	1.94 (1.16,3.25)	0.01	66%	0.05
CA for BPD	8	61558	3.18 (1.68,6.03)	0.0004	93%	< 0.00001
^d^HCA for BPD	7	6005	2.70 (2.38,3.07)	< 0.00001	0%	0.79
HCA for mild BPD	1	184	2.29 (0.99,5.31)	0.05	NA	NA
HCA for moderate to severe BPD	1	139	25.38 (7.13,90.32)	< 0.00001	NA	NA
^e^CCA for BPD	2	59631	2.77 (0.68,11.21)	0.15	99%	< 0.00001
FV for BPD	1	231	6.36 (2.45,16.52)	0.0001	NA	NA

^a^*BPD* Bronchopulmonary dysplasia

^b^*CA* Chorioamnionitis

^c^*FV* Funisitis

^d^*HCA* Histological chorioamnionitis

^e^*CCA* Clinical chorioamnionitis

#### Different severity degrees of BPD

BPD is classified as mild, moderate, severe, and moderate or severe. As a result of the data collected, we concluded that infants exposed to HCA have an increased risk of developing moderate to severe BPD (OR = 25.38, 95%CI: 7.13–90.32), whereas no obvious meaningful link has been demonstrated between HCA and mild BPD (OR = 2.29, 95%CI: 0.99–5.31). And no research has been conducted on moderate and severe BPD.

### High-quality studies

Additional file 3 summarizes the quality of each study according to the Newcastle–Ottawa Scale. Out of a possible nine points, studies received a quality score of 6 points (2 studies), 7 points (9 studies), or 8 points (5 studies). In total, we obtained 14 high-quality studies (at least 7 points) [19, 31–37, 39–42, 44], and the results showed statistical significance (OR = 1.49, 95%CI: 1.09–2.03, I^2^ = 96%) (Table 1).

### Publication bias

**Fig. 4:**
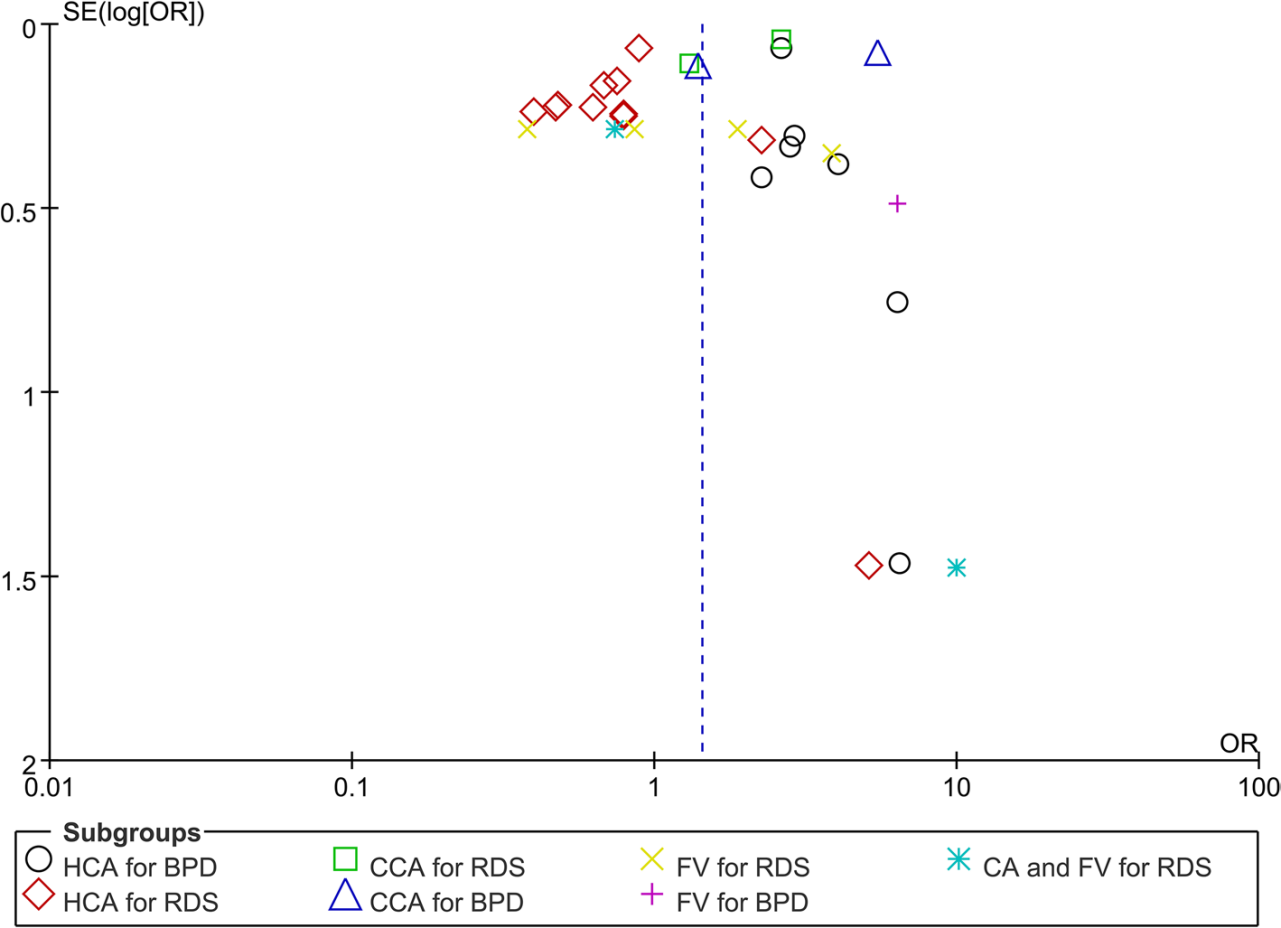
Funnel plot of Publication bias of visual inspection

Publication bias of visual inspection of funnel plots, concentrated and symmetrical, is reported in Fig. 4. Neither the Begg’s test (*P* > 0.05) nor visual inspection of funnel plots indicated publication or selection bias. There were insufficient studies with different stages of HCA for NRDS and different severity degrees of BPD to evaluate publication bias.

## Discussion

In the 1990s, Watterberg et al. [46] first discovered that intrauterine infection had a protective effect on NRDS. In some subsequent studies, the same effect was observed [47, 48], which were attributed the effect to inflammation promoting the generation of PS and fetal lung maturation [38, 49–51]. Ding Ran et al. [42] found that in the HCA-exposed group, NRDS incidence was only 25.5%, significantly lower than 45.9% in the control group, consistent with previous findings. Despite the fact that many animal experiments and clinical studies have demonstrated that intrauterine inflammation has a potential protective effect against the occurrence of NRDS, this view has not been unanimously recognized. Gisslen et al. [52] found that the increase in interleukin-6 in cord blood could not reduce the incidence of NRDS in infants. Some studies also found that intrauterine infection could not [18, 20, 51–53].

In our meta-analysis, we found no correlation between CA/FV (CA, FV, CA with FV and CCA) and NRDS, but HCA could reduce NRDS risk and is more likely to occur in preterm infants less than 32 weeks of gestation. The protective effect exists in developing countries, but not in developed countries, which may be related with their economic situation, medical development level, living environment, etc. A possible explanation for the protective effect may be that, during intrauterine infection, saturation phosphatidylcholine and surfactant active protein B (SP-B), which regulate lung tension and maintain the physiological state of the lungs, are produced at higher levels. Moreover, some studies have shown that inflammation increases levels of interleukin-6 in fetal peripheral blood, which in turn triggers the production of SP-A in the alveolar epithelium and ultimately increases the production of PS [18, 50, 51]. Additionally, premature infants under 32 weeks of gestation are in the late tubule or cystic stage, with less PS production itself and little protective effect on the fetal lungs, while the role of promoting fetal lung maturation through intrauterine inflammation is more obvious. Furthermore, it is debatable whether severe HCA increases or decreases the incidence of NRDS. Some scholars believe that fetal lung maturity can only be promoted if inflammation is not severe, and severe inflammation can damage fetal lung tissue. Severe inflammation may increase vascular permeability in fetal lungs, leading to PS inactivation as serum proteins leak into alveoli [54–56]. Our findings indicate that infants exposed to HCA (I) and (II) had a reduced incidence of NRDS, referred to as a “protective effect”, while HCA (III) was not associated with NRDS. This is somewhat different from the Waterberg hypothesis “early-protection, late-damage effect”. We could not exclude the bias that may caused by the data provided by only 3 studies. For different grade of NRDS, no study provided relevant data.

A recent meta-analysis of studies involving 13,583 infants demonstrated that histological, as opposed to clinical chorioamnitis, was associated with a higher incidence of BPD [23]. The association was no longer significant after accounting for the lack of unpublished studies showing an association and the potential publication bias, including gestational age and birth weight [23]. ELGAN [57] reported no association between HCA and BPD, although 51% of placental cultures obtained from 1,119 extremely premature births produced positive results. Nor was a relationship between placental signs of cellular inflammation and BPD observed in the Alabama preterm birth study, although umbilical blood cultures positive for ureaplasma urealyticum or Mycoplasma hominis more than tripled the risk of BPD (26.8% vs. 10.1%, *p* = 0.0001) [58, 59]. However, in multivariate analysis, this association was not strong (OR 1.99, 95%CI 0.91–4.37) [59]. We find that CA/FV increased the incidence of BPD, regardless of the diagnosis of BPD (BPD28, BPD36). In addition, HCA was associated with BPD, while CCA was not. This may be due to the fact that only two studies provide data on CCA and BPD. There is no data available on the different stages of HCA and BPD. Upon categorizing BPD, only moderate to severe BPD was related to CA, while the other grades were not. Bias may be caused by insufficient data. Otherwise, the inclusion criteria of this study were CA as the exposure factor and BPD or absence of BPD in preterm neonates as the outcome. And some regression studies were excluded: the outcome had been BPD in preterm infants, and the risk factors were retrospectively traced back. Because we were unable to obtain data from these studies on preterm infants exposed to CA who did not develop BPD, controls could not be performed. Moreover, some studies cannot exclude the influence of other factors such as gestational hypertension, diabetes and other complications on the occurrence of BPD in preterm infants. Therefore, we only included cohort studies with higher levels of evidence to reduce the bias. This is one of the different points of inclusion criteria in the study published in 2019 by Villamor-Martinez et al. [22], that one of the meta-analyses of related studies.

In their study published in 2012, Hartling et al. [21] only discussed the relationship between CA and BPD based on English literature. Although the data revealed a significant correlation between CA and BPD (OR 1.89, 95%CI 1.56–2.3), there was still a substantial amount of bias. CA is not explicitly acknowledged as a BPD risk factor. In the literature published by Sarno et al. [23] in 2021, we found that the inclusion criteria for chorionicamnitis were HCA, excluding CCA, and the data showed that there was no statistical correlation between HCA and NRDS (RR 0.93, 95%CI 1.08–1.67). Nevertheless, it was positively correlated with BPD (RR 1.75, 95%CI 1.37–2.23). After excluding the effect of preterm birth, it was found that HCA could reduce the incidence of NRDS (RR 0.57, CI 95% 0.35–0.93), but had no statistical significance with BPD (RR 0.99, CI 0.76–1.3). Preterm infants were not analyzed separately in this study. And the incidence of BPD and NRDS was not significantly correlated with HCA or FV. Villamor-Martinez et al. [22] published in 2019 confirmed that exposure to CA is associated with a higher risk of developing BPD among preterm infants, but this association may also be modulated by gestational age and risk of NRDS.

Compared to the previous systematic review [21–23], this study includes a substantial update; the term funisitis has been included in our search strategy; detailed grading of exposures and outcomes has been performed; and multiple risk factors for BPD and NRDS have been excluded from the study. With the advancement of science, the definition of CA/BPD/NRDS has become more precise. In order to avoid the bias caused by different definitions, we excluded studies where explicit definitions were not provided and selected literature based on the same definition, instead of the time line, which differed from Sarno et al. [23]. Moreover, at present, there is no systematic review about this subject in China, on this basis, our study on the relationship between CA and lung injury in preterm infants compares the differences between developed and developing countries. There are some limitations in this study. First, the data collection is not comprehensive and lacks unpublished literature, which may result in the omission of some positive or negative results, thereby introducing bias into the meta-analysis. Additionally, the number of studies on the relationship between different stages of HCA and different degrees of lung injury in preterm infants is limited, which may affect the bias of the meta-analysis results the number of studies on the relationship between different stage of HCA and different degree lung injury in preterm infants is small, which may affect the bias of the meta-analysis results. Due to limited conditions, our study on the relationship between CA and lung injury in preterm infants did not compares the differences among ethnic groups, environmental factors, and genetic factors. Finally, this study does not assess whether the effect of CA on lung injury in premature infants was gender-specific because of the paucity of pertinent data.

## Conclusions

Overall, the current evidence suggests that CA or FV appears to be associated with a higher incidence of lung injury in preterm infants, particularly in developed countries. And HCA can increase the incidence of BPD but reduce the incidence of NRDS. We agree with the viewpoint of ‘early protection’, however, we disagree with ‘late damage’, which requires further clinical research and observation. In addition, the results of this study indicate that this “protective effect” only applies to infants under 32 weeks of age. Furthermore, the incidence of BPD can also be increased by HCA, highlighting the importance of improving the attention of clinical workers to premature infants and requesting NICU consultation as soon as possible after birth in order to initiate early intervention and guide a later diagnosis and treatment for such premature infants. What’s more, a further study is necessary to clarify the role of CA in the severity of lung injury in preterm infants and to exclude the effect of gestational age itself on lung injury.

### Supplementary Information

**Supplementary Material 1. **

**Supplementary Material 2. **

**Supplementary Material 3. **

## Data Availability

All data generated or analyzed during this study are included in this article and its online supplementary material. Further inquiries can be directed to the corresponding author.

## Abbreviations

CA: Chorioamnionitis
HCA: Histological chorioamnionitis
CCA: Clinical chorioamnionitis
FV: Funisitis
NRDS: Neonatal respiratory distress syndrome
BPD: Bronchopulmonary dysplasia
