# Supplementary material for: Relationship between chorioamnionitis or funisitis and lung injury among preterm infants: meta-analysis involved 16 observational studies with 68,397 participants
Source: BMC Pediatr. 2024 Mar 5;24:157. doi: 10.1186/s12887-024-04626-0 (PMC10916086; doi:10.1186/s12887-024-04626-0)
Supplement: Supplementary file 1 — Supplementary Material 1. [file 12887_2024_4626_MOESM1_ESM.docx]

**Additional file 1.** Characteristics of included studies

| **Study, Year** | **Country** | **Design** | **Included patients** | **Mean±SD, Age Range of Cases, W** | **Mean±SD, Weight Range of Cases, g** | | **Outcome** | | | **Definition of BPD** | **Exposure** | | **Non-Exposure** | |  |
| --- | --- | --- | --- | --- | --- | --- | --- | --- | --- | --- | --- | --- | --- | --- | --- |
| Zhang,H, 2022 | China | retrospective | 200 | BPD:29.25±1.99,  N-BPD:30.93±1.10 | | BPD:1487.27±314.98, N-BPD:1595.88±318.29 | | BPD | At 28 days after birth, with any oxygen dependence (oxygen concentration> 21%), gestational age <32 weeks, BPD children were divided into mild BPD (oxygen concentration <30%), moderate to severe BPD (moderate oxygen concentration of 30%~40%, severe oxygen concentration> 40%, or still need device ventilation) | | | HCA | | N-HCA | |
| Zhang,K, 2022 | China | prospective | 317 | HCA:28.9 (30.7, 32.9),  N-HCA:31.9 (31.0, 32.6) | | HCA:1681.5±382.8,  N-HCA:2018.3±334.0 | | RDS | NA | | | HCA | | N-HCA | |
| DING, 2021 | China | retrospective | 376 | HCA: the early: 30.4 (28.9, 31.1),  the mid: 29.7 (28.7, 30.9),  the late: 30.1 (29.3, 30.5),  N-HCA: 30.7 (28.9, 31.4） | | HCA: the early: 1477±307,  the mid: 1452±274,  the late: 1392±175 | | RDS | NA | | | HCA | | N-HCA | |
| Fang, 2021 | China | retrospective | 689 | HCA: 30.9(29.1, 32.9),  N-HCA: 32.3(31.0, 33.3) | | HCA: 1570(1290, 1967),  N-HCA: 1720(1390, 2020) | | RDS/BPD | The infants remained oxygen dependent beyond 28 days, they were diagnosed at 36 weeks PMA to have mild BPD (no longer oxygen dependent), moderate BPD (required less than 30% oxygen) or severe BPD (required more than 30% oxygen and/or positive pressure support) | | | HCA | | N-HCA | |
| Zhang, 2020 | China | retrospective | 361 | HCA: 31.3±2.0,  N-HCA: 31.7±1.8 | | 1742±308 | | BPD | At 28 days after birth, with any oxygen dependence (oxygen concentration> 21%), gestational age <32 weeks, BPD children were divided into mild BPD (oxygen concentration <30%), moderate to severe BPD (moderate oxygen concentration of 30%~40%, severe oxygen concentration> 41%, or still need device ventilation) | | | HCA | | N-HCA | |
| Metcalfe, 2017 | Canada | retrospective | 56537 | CCA: 31.1,  N-CCA: 34.2 | | CCA: 2086.9,  C-CCA: 2587.3 | | RDS/BPD | Followed the Eunice Kennedy Shriver National Institute of Child Health and Human Development consensus definition | | | CCA | | N-CCA | |
| Xie, 2017 | China | prospective | 151 | 31.3±1.7  (HCV(+)FV(+): 31.1±1.7,  HCV(+)FV(-): 31.7±1.8,  HCV(-)FV(-): 31.9±1.2) | | 1731.7±384.7  (HCV(+)FV(+): 1685.6±386.9, HCV(+)FV(-): 1814.9±430.5, HCV(-)FV(-): 1765.2±339.5) | | RDS/BPD | At 28 days after birth, with any oxygen dependence (oxygen concentration> 21%), gestational age <32 weeks, BPD children were divided into mild BPD (oxygen concentration <30%), moderate to severe BPD (moderate oxygen concentration of 30%~40%, severe oxygen concentration> 41%, or still need device ventilation) | | | HCV/FV | | N-HCA/N-FV | |
| Cai, 2016 | China | retrospective | 712 | HCA: mild: 32.8±1.5,  moderate: 30.1±2.1,  severe: 29.8±1.8,  N-HCA: 32.9±1.6 | | HCA: mild: 1818±401,  moderate: 1568±331,  severe: 1518±361,  N-HCA: 1838±431 | | RDS | NA | | | HCA | | N-HCA | |

**Continued**

| **Study, Year** | **Country** | **Design** | **Included patients** | | **Mean±SD, Age Range of Cases, W** | **Mean±SD, Weight Range of Cases, g** | **Outcome** | **Definition of BPD** | **Exposure** | **Non-Exposure** |
| --- | --- | --- | --- | --- | --- | --- | --- | --- | --- | --- |
| Li, 2016 | China | retrospective | 295 | HCA: 32±2.8,  N-HCA: 32±2.8 | | HCA: 1730.2±424，,  N-HCA: 1630.7±416 | RDS/BPD | The infants remained oxygen dependent beyond 28 days, they were diagnosed at 36 weeks PMA to have mild BPD (no longer oxygen dependent), moderate BPD (required less than 30% oxygen) or severe BPD (required more than 30% oxygen and/or positive pressure support) | HCA | N-HCA |
| Miyazaki, 2016 | Japan | retrospective | 4078 | HCA: 26.5±2.6,  N-HCA: 28.1±2.8 | | HCA: 921±295,  N-HCA: 995±302 | RDS/BPD | Dependency on oxygen supplementation at a corrected age of 28 d | HCA | N-HCA |
| Park, 2015 | Korea | prospective | 378 | HCA: minimal: 32.4(24.6, 34.0),  mild: 30.9(24.7, 34.0),  moderate: 29.4(24.7, 34.0),  severe: 29.7(24.9, 34.0),  N-HCA: 32.1(25.3, 34.0) | | HCA: minimal: 1708±482,  mild: 1587±502,  moderate: 1468±483,  severe: 1380±453,  N-HCA: 1792±467 | RDS | NA | HCA/FV | N-HCA/N-FV |
| Zhang, , 2015 | China | retrospective | 347 | HCA: 30.05±1.07,  N-HCA: 29.98±1.04 | | HCA: 1685±420,  N-HCA: 1702±409 | RDS | NA | HCA/FV | N-HCA/N-FV |
| Tsiartas, 2013 | Sweden | prospective | 231 | HCA: 32+3,  N-HCA: 34+0;  FV: 31,  N-FV: 33 | | HCA: 1840,  N-HCA: 2190 | RDS/BPD | Oxygen dependency at 28 d of life | HCA/FV | N-HCA/N-FV |
| Lee, 2011 | Korea | retrospective | 301 | FV: 29 (24.1-32),  N-FV: 29.4 (24-32) | | FV: 1245 (530-2010),  N-FV: 1160 (410-2360) | RDS | NA | FV | N-FV |
| Soraisham, 2009 | Canada | retrospective | 3094 | CCA: 27.7±2.7,  N-CCA: 29.1±2.5 | | CCA: 1174±439,  N-CCA: 1347±460 | RDS/BPD | Supplemental oxygen dependency at 36 weeks of corrected gestational age oxygen | CCA | N-CCA |
| Dempsey, 2005 | Canada | retrospective | 330 | HCA: 26.3±2,  N-HCA: 27.5±1.9 | | HCA: 920±284,  N-HCA: 1030±357 | RDS | NA | HCA | N-HCA |
