# Supplementary material for: Relationship between chorioamnionitis or funisitis and lung injury among preterm infants: meta-analysis involved 16 observational studies with 68,397 participants
Source: BMC Pediatr. 2024 Mar 5;24:157. doi: 10.1186/s12887-024-04626-0 (PMC10916086; doi:10.1186/s12887-024-04626-0)
Supplement: Supplementary file 2 — Supplementary Material 2. [file 12887_2024_4626_MOESM2_ESM.docx]

**Additional file 2.** The reasons of exclude literature

| **Study** | **Year** | **Title** | **Reasons** |
| --- | --- | --- | --- |
| Budal | 2022 | Placental histology predicted adverse outcomes in extremely premature neonates in Norway-population-based study | Incomplete data |
| Cai | 2022 | Correlation between premature rupture of membranes with chorioamnionitis and respiratory distress syndrome in very preterm infants | Incomplete data |
| Liu | 2021 | Analysis of maternal high risk factors for neonatal acute respiratory distress syndrome | The participants does not meet inclusion criteria |
| Sharma | 2021 | Chronic Inflammatory Placental Lesions Correlate With Bronchopulmonary Dysplasia Severity in Extremely Preterm Infants | Incomplete data |
| Chen | 2021 | Gestational age &lt; Risk factors of bronchopulmonary dysplasia in 32-week preterm infants | Inconsistent research method |
| Liu | 2021 | Level and clinical significance of serum inflammatory markers in premature infants with bronchopulmonary dysplasia | Inconsistent research method |
| Zhang | 2021 | Construction of early risk prediction model for bronchopulmonary dysplasia in premature infants | Inconsistent research method |
| Ryan | 2020 | Histological chorioamnionitis is predicted by early infant C-reactive protein in preterm infants and correlates with neonatal outcomes | The participants does not meet inclusion criteria |
| Lee | 2020 | Risk of intra-amniotic infection/inflammation and respiratory distress syndrome according to the birth order in twin preterm neonates | Other reasons |
| Jackson | 2020 | Pulmonary Consequences of Prenatal Inflammatory Exposures: Clinical Perspective and Review of Basic Immunological Mechanisms | Other reasons |
| Humberg | 2020 | Preterm birth and sustained inflammation: consequences for the neonate | Review |
| Bancalari | 2020 | Antenatal Infections and Respiratory Outcome in Preterm Infants | Review |
| Budal | 2020 | Histological chorioamnionitis in placentas of extremely premature neonates: The impact of maternal and foetal inflammatory responses on clinical findings and neonatal outcome | Nonoriginal articls |
| Liu | 2020 | Analysis of influencing factors of bronchopulmonary dysplasia in very premature infants | Inconsistent research method |
| Yang | 2020 | Risk factors analysis of bronchopulmonary dysplasia in premature infants | Inconsistent research method |
| Zou | 2020 | Risk factors and early prediction of bronchopulmonary dysplasia in very premature infants | Inconsistent research method |
| Zhang | 2019 | Clinical analysis of histological diagnosis of chorioamnionitis and maternal and infant outcomes in cases of premature rupture of membranes before 34 weeks of gestation | The participants does not meet inclusion criteria |
| Wang | 2019 | Correlation analysis between patients with premature rupture of membranes and subclinical chorioamnionitis and pregnancy outcomes | The participants does not meet inclusion criteria |
| Huo | 2019 | Analysis of influencing factors of bronchopulmonary dysplasia in very low birth weight infants | The participants does not meet inclusion criteria |
| Pinto | 2019 | Neonatal outcome in preterm deliveries before 34 week gestation-the influence of the mechanism of labor onset | Exposure does not match |
| **Continued** |  |  |  |
| **Study** | **Year** | **Title** | **Reasons** |
| Pietrasanta | 2019 | Impact of different stages of intrauterine inflammation on outcome of preterm neonates: Gestational age-dependent and -independent effect | The participants does not meet inclusion criteria |
| Lee | 2019 | Histological chorioamnionitis, antenatal steroids, and neonatal outcomes in very low birth weight infants: A nationwide study | The participants does not meet inclusion criteria |
| Dang | 2019 | Analysis of risk factors of bronchopulmonary dysplasia in 20 premature infants | Inconsistent research method |
| Yang | 2019 | The incidence and related factors of bronchopulmonary dysplasia in premature infants from 2015 to 2018 | Inconsistent research method |
| Yang | 2019 | Analysis of risk factors of bronchopulmonary dysplasia in premature infants | Inconsistent research method |
| Zhang | 2019 | Analysis of high risk factors of bronchopulmonary dysplasia in premature infants | Inconsistent research method |
| Zhang | 2019 | Follow-up analysis of high risk factors of bronchopulmonary dysplasia and wheezing in infancy | Inconsistent research method |
| Liu | 2018 | Relationship between placental histologic chorioamnionitis and preterm labor outcome | The participants does not meet inclusion criteria |
| Bhunwal | 2018 | Bronchopulmonary Dysplasia in Preterm Neonates in a Level III Neonatal Unit in India | Inconsistent research method |
| Sang | 2018 | Clinical analysis of respiratory distress syndrome in premature infants | Inconsistent research method |
| Liang | 2017 | Relationship between latency period and pregnancy outcome in 58 cases of preterm premature rupture of membranes | Exposure does not match |
| Guan | 2017 | Correlation between premature rupture of membranes and subclinical chorioamnionitis and pregnancy outcomes | The participants does not meet inclusion criteria |
| Feng | 2017 | High risk factors of placental chorioamnionitis and the correlation with neonatal adverse outcomes | The participants does not meet inclusion criteria |
| Nobile | 2017 | Neonatal outcome of small for gestational age preterm infants | Exposure does not match |
| Catov | 2017 | Neonatal outcomes following preterm birth classified according to placental features | The outcome does not meet inclusion criteria |
| Maksic | 2017 | Risk factors for bronchopulmonary dysplasia in very preterm infants | Inconsistent research method |
| Torchin | 2017 | Histologic Chorioamnionitis and Bronchopulmonary Dysplasia in Preterm Infants: The Epidemiologic Study on Low Gestational Ages 2 Cohort | Inconsistent research method |
| WU Ying | 2016 | Clinical analysis of histological diagnosis of chorioamnionitis and maternal and infant outcomes in cases of premature rupture of membranes before 34 weeks of gestation | Incomplete data |
| WU Pan | 2016 | Analysis of related factors of neonatal intrauterine infectious pneumonia in patients with premature rupture of membranes | The outcome does not meet inclusion criteria |
| Yang | 2016 | Clinical study of 200 premature infants with bronchopulmonary dysplasia | Inconsistent research method |
| **Continued** |  |  |  |
| **Study** | **Year** | **Title** | **Reasons** |
| Zhang | 2015 | Discussion on Predisposing Factors of Patients with Preterm Premature Rupture of the Membranes and Pregnancy Outcome | Exposure does not match |
| Yuan | 2015 | Effect of subclinical chorioamnionitis on pregnancy outcome | The participants does not meet inclusion criteria |
| Qi | 2015 | Logistic regression analysis of risk factors for bronchopulmonary dysplasia complicated with pulmonary hypertension | The outcome does not meet inclusion criteria |
| Xie | 2015 | Related factors and adverse neonatal outcomes in women with preterm premature rupture of membranes complicated by histologic chorioamnionitis | Exposure does not match |
| Kim | 2015 | Neonatal Morbidities Associated with Histologic Chorioamnionitis Defined Based on the Site and Extent of Inflammation in Very Low Birth Weight Infants | The participants does not meet inclusion criteria |
| Ericson | 2015 | Chorioamnionitis: implications for the neonate | The outcome does not meet inclusion criteria |
| Anuk | 2015 | Impact of placental histopathology and weight on neonatal outcomes in preterm infants | Nonoriginal articls |
