# Supplementary material for: Relationship between chorioamnionitis or funisitis and lung injury among preterm infants: meta-analysis involved 16 observational studies with 68,397 participants
Source: BMC Pediatr. 2024 Mar 5;24:157. doi: 10.1186/s12887-024-04626-0 (PMC10916086; doi:10.1186/s12887-024-04626-0)
Supplement: Supplementary file 3 — Supplementary Material 3. [file 12887_2024_4626_MOESM3_ESM.docx]

| **Additional file 3.** Quality Assessment of Included Studies in the Meta-analysis Using the NOS | | | | | | | | | |
| --- | --- | --- | --- | --- | --- | --- | --- | --- | --- |
| Source | Representativeness of the exposed cohort | Selection of the non-exposed cohort | Ascertainment of exposure | Demonstration that outcome of interest was not present at start of study | Comparability of cohorts on the basis of the design or analysis | Assessment of outcome | Was follow-up long enough for outcomes to occur | Adequacy of follow-up of cohorts | Total  NOS  Score |
| Cai 2016 | * | * | * |  | * | * | * | * | 7 |
| Dempsey 2005 | * | * | * |  | ** | * | * | * | 8 |
| Ding 2021 | * | * | * |  | ** | * | * | * | 8 |
| Fang 2021 | * | * | * |  | * |  | * | * | 6 |
| Lee 2011 | * | * | * |  | ** | * | * | * | 8 |
| Li 2016 | * | * | * |  | ** | * | * | * | 7 |
| Metcalfe 2017 | * | * | * |  | ** | * | * | * | 8 |
| Miyazaki 2016 | * | * | * |  | * | * | * | * | 7 |
| Park 2015 | * | * | * | * | * |  | * | * | 7 |
| Soraisham 2009 | * | * | * |  | * | * | * | * | 7 |
| Tsiartas 2013 | * | * | * | * | * |  | * | * | 7 |
| Xie 2017 | * | * | * | * | * |  | * | * | 7 |
| Zhang,H 2022 | * | * | * |  | ** | * | * | * | 8 |
| Zhang,K 2022 | * | * | * |  | * |  | * | * | 6 |
| Zhang,L 2015 | * | * | * | * | * |  | * | * | 7 |
| Zhang,L 2020 | * | * | * |  | ** |  | * | * | 7 |
